# Dietary inflammatory index is not associated with bone mineral density in functionally able community-dwelling older adults

**DOI:** 10.1007/s00394-024-03500-0

**Published:** 2024-09-24

**Authors:** Corey Linton, Mia A. Schaumberg, Hattie H. Wright

**Affiliations:** 1 School of Health, University of the Sunshine Coast, Sippy Downs, Australia; 2 Sunshine Coast Health Institute, Birtinya, Sunshine Coast Australia; 3 Manna Institute, University of the Sunshine Coast, Birtinya, QLD Australia; 4 School of Human Movement and Nutrition Sciences, The University of Queensland, Brisbane, Australia

**Keywords:** Diet, Inflammation, Ageing, Bone health, Osteoporosis

## Abstract

**Background:**

Osteoporosis poses a significant health and quality-of-life burden on older adults, particularly with associated fractures after a fall. A notable increase in pro-inflammatory cytokines associated with aging contributes to a decline in bone mineral density (BMD). Certain food components have been shown to influence an individual’s inflammatory state and may contribute to optimal bone health as a modifiable risk factor, particularly later in life. This study aims to explore the relationship between the dietary inflammatory index (DII) and dietary intake with BMD in community-dwelling older adults.

**Methods:**

Heathy community-dwelling older adults aged 65–85 years. DII scores were calculated using 24-h dietary recalls, and lumbar spine (L1–L4) and femoral neck (ward’s triangle) BMD was assessed via dual-energy x-ray absorptiometry.

**Results:**

A total of 94 participants were recruited (72.9 ± 4.9 years, 76.6% female) with 61.7% identified having an anti-inflammatory diet (average DII = − 0.50 ± 1.6), 88.3% were physically active, 47.8% were osteopenic and 27.7% osteoporotic. There was no significant difference between DII scores, nutrient or food group intake in groups stratified by BMD T-Score except for lean meats and alternatives food group (p = 0.027). Multiple regression analysis found no associations between DII and lumbar spine (unadjusted model β = 0.020, p = 0.155) or femoral neck BMD (unadjusted model β = − 0.001, p = 0.866).

**Conclusion:**

Most of this cohort of functionally able community-dwelling older adults followed an anti-inflammatory diet. DII and dietary intake were not associated with BMD. This research underlines the complex interplay between modifiable and non-modifiable risk factors on the BMD of older, physically active adults.

## Introduction

Osteoporosis is defined as the loss of bone mineral density (BMD) or the deterioration of bone tissue and can increase the risk of bone fractures from a fall, which may result in reduced quality of life and increased health care costs [1, 2]. Osteoporosis is prevalent among older adults, particularly women older than 60 years [1]. It is estimated that osteoporosis and associated fractures currently cost the global health care system between $5 and $6.5 trillion USD per year [3]. Osteoporosis not only imposes a significant financial burden on the health care system but also adversely affects health-related quality of life, by potentially diminishing their independence and mobility of older adults [2]. As the population ages and life expectancy continues to rise, the incidence of osteoporotic fractures, particularly in elderly individuals, is projected to increase by 310% in men and 240% in women by 2050 [4]. The development of osteoporosis is influenced by several genetic, hormonal and lifestyle factors, collectively contributing to its intricate pathophysiology [5]. Regardless of contributing factors, osteoporosis is caused by an imbalance of bone resorption in excess of bone formation [6]. This bone remodelling is a normal physiological process which sees old bone removed by osteoclasts, the process known as bone resorption, and new bone is formed by osteoblasts, known as bone formation [7]. Into older age this process tends to favour bone resorption rather than bone formation and BMD decreases [6, 7]. Ageing sees a detrimental effect on the regulation and maintenance of apoptosis which results in increased death of osteocytes, alterations to bone homeostasis, and in turn the decline in BMD [8]. This typical decline in BMD through age-associated nonheritable risk factors results in an increased risk of fracture into later years.

The decline in BMD is accelerated by many modifiable risk factors such as sedentary behaviour, dietary choices, cigarette smoking, and inflammation [7, 9]. Inflammation plays a pivotal role in the progression of osteoporosis, as chronic inflammation significantly impacts bone metabolism, resulting in an elevated resorption rate and an increased risk of fractures [10]. Briefly, a notable increase in pro-inflammatory cytokines associated with aging augments myelopoiesis and osteoclastogenesis, thereby contributing to a decline in BMD [11]. Inflammatory cytokines have been found to shift the bone remodelling process to resorption, where elevated interleukin-6 (IL-6), a precursor to CRP, has been associated with a decrease in BMD over time [13]. Evidence suggests there are many pro-inflammatory cytokines (IL-1, TNF-α, IL-6, IL-11, IL-15 and IL-17) that are capable of stimulating osteoclastic bone resorption ultimately resulting in a decrease in BMD [11]. Specifically, TNF-α promotes systemic receptor activator of nuclear factor-kappa ligand production alongside IL-1 which acts on osteoblasts inducing Prostaglandin E2, inducing osteoclast formation and a reduction in BMD [12, 14]. Physical activity is a key modifiable risk factor for bone health and regular physical activity is known to increase or maintain BMD into later life [15]. Regular physical activity induces mechanical pressure onto the entire musculoskeletal system which results in strengthening of bone as well as reducing levels of oxidative stress throughout the body [16, 17]. Osteocytes sense the mechanical load of exercise which signals cells at the bone surface triggering bone remodelling, osteocytes decrease their secretion at this time resulting in bone remodelling process favouring bone formation [17]. The increase in mechanical load from physical activity, decreases secretion of sclerostin by osteocytes, resulting in an elevated activation of the Wnt–β-catenin signalling pathway, resulting in increased bone formation as well as improvements in BMD and bone strength [7].

Diet is one of several modifiable risk factors which can contribute to achieving and maintaining optimal bone health across the lifespan [18, 19]. Key macro- and micro-nutrients have been well described to play a role in the increase and preservation of BMD [20–22]. Further, it is now widely acknowledged that the combined influence of nutrients (such as protein, calcium, vitamin D, and potassium), non-nutrients (e.g., phytochemicals), and food components (e.g., vegetables, fruit, lean meat, and meat alternatives) can positively influence health outcomes, including bone health [23, 24]. The recognition of this synergistic dietary effect has resulted in a paradigm shift away from single nutrient research to examining the effect of diet as a whole on health outcomes [23, 25]. Dietary patterns are used to examine the health benefits of whole diets through posteriori or a priori methodologies [26]. The posterior approach uses empirical data and statistical methods to generate a dietary pattern unique to a population while the a priori approach use existing knowledge to develop a predefined dietary pattern associated with specific health outcomes [26]. The dietary inflammatory index (DII) is an a priori method designed to evaluate the inflammatory potential of a diet [27].

There is clear evidence supporting the influence of certain foods on inflammatory biomarkers, such as fruits, vegetables, nuts and whole grains that are able to reduce inflammatory biomarkers [28]. On the other hand, processed foods and animal products are associated with higher concentrations of CRP and IL-6, both of which are pro-inflammatory cytokines [28–30]. Despite evidence linking diet-induced inflammation and its role in the progression of osteoporosis, research investigating the direct association between the inflammatory potential of the diet and BMD has reported mixed findings [31–38]. Therefore, this study aims to firstly examine the relationship between DII and BMD in a cohort of community-dwelling older adults and secondly to explore the associations between literature determined nutrients and food groups on BMD.

## Materials and methods

### Participants

The present study was a sub-study of a larger community evaluation project which recruited apparently healthy, independent community-dwelling older adults, ranging in age from 65 to 85 years, who possessed the physical capability to engage in daily activities [39]. This recruitment effort was conducted in collaboration with the local government as part of a comprehensive community-based evaluation project. The recruitment strategy encompassed multiple avenues, including outreach through community exercise classes, dissemination via email lists, online advertising, informative presentations, and referrals through personal networks. The inclusion criteria for this study were community-dwelling older adults (aged 65 years and older), residing in the Sunshine Coast Local Government Area of Queensland, Australia and participants were classified as ‘not high risk of experiencing a cardiac event during exercise’ according to the adult pre-exercise screening system [40]. Individuals with medical conditions such as uncontrolled hypertension, cardiomyopathy, unstable angina, heart failure, severe arrhythmia, cancer, or chronic communicable infectious diseases were excluded. This research received ethical approval from the Institutional Human Research Ethics Committee (#A201498).

#### Assessment of dietary intake

An accredited practicing dietitian collected three, 24-h dietary recalls (24 h recall) from each participant via phone or in person over a two-week period following each individual in-person DXA scan using the multiple pass method [41]. There was a minimum of 2 days between each 24 h recall which aimed to incorporate two weekdays and one weekend day. Data collection took place over 18-months resulting in all seasons being represented across the cohort. Food portion size was evaluated using standardised serving sizes and household measures, and participants were provided a food model booklet to assist in quantifying food and beverage intake. Habitual dietary intake was calculated from the average of the three, 24 h recalls which were manually entered in FoodWorks^®^ Professional Version 10. FoodWorks^®^ is an Australian nutrient analysis software program which utilises the AUSNUT 2011–2013 food composition database [42]. Energy, nutrient, and food group intake were exported into Excel prior to SPSS analysis. Over- and under-reporting was assessed according to the Goldberg ratio method (reported energy intake to basal metabolic rate, EI:BMR) [43, 44]. Cut-points included EI:BMR > 2.62 for men and > 2.42 for women as over-reporting and < 1.21 for men and < 1.11 for women as under-reporting [43]. Basal metabolic rate was calculated using the Mifflin St Jeor equation [45]. Both under- and over-reporters were excluded from dietary analysis.

#### Calculation of dietary inflammatory index (DII) scores

Dietary data from FoodWorks^®^ was imputed into the developed DII calculation tool to determine the inflammatory potential of the diet. A detailed description on the development of the DII is provided elsewhere [46, 47] however, in short the DII was developed to assess the inflammatory potential of the diet; allowing researchers to identify both pro- and anti-inflammatory diets [46]. The DII scoring methodology was constructed by collating data from peer-reviewed research articles reporting the influence of specific foods and nutrients on circulating inflammatory markers [46]. The cumulative score from this tool captures the combined effect of dietary components on inflammation, where a more negative score indicates an anti-inflammatory diet and a more positive score indicates a pro-inflammatory dietary pattern with a potential score ranging from − 8.87 to 7.98 [46]. Garlic, ginger, saffron, turmeric, pepper, thyme/oregano, and rosemary were not included in calculating the DII score due to the difficulty in quantifying portion sizes. In addition, items not calculated by FoodWorks^®^ namely vitamin D, flavan-3-ol, flavones, flavanols, flavanones, anthocyanidins and isoflavones were not included. Others have also excluded similar foods and non-nutrients from calculated DII scores due to quantification difficulty [34, 36, 38, 48].

### 2.3. Assessment of anthropometry

Height (Harpeden Wall Mounted Stadiometer), body mass (A&D HW-200KGL Calibrated Scales), and waist circumference (Cescorf Tape Measure) were measured following standard International Society for the Advancement of Kinanthropometry (ISAK) procedures. To calculate body mass index (BMI) the body mass relative to squared height was used [49]. Underweight (BMI < 23 kg/m^2^), normal weight (BMI = 24–30 kg/m^2^) and overweight (> 30 kg/m^2^) were determined using standard BMI cut-offs for adults over the age of 65-years [50].

### 2.4. Measurement of bone mineral density (BMD)

Lumbar spine (L1–L4) and femoral neck (wards triangle) BMD (g/cm^2^) were determined using dual-energy x-ray absorptiometry (DXA, GE Lunar iDXA, GE encore software version 13.60) in accordance with the manufacturer’s instructions and the protocol developed by Nana and colleagues [51]. Both individual scans were conducted between 6–8 am, participants were overnight fasted, rested and euhydrated prior to the scan. All participants removed metal objects and jewellery from their body and wore minimal clothing to ensure all scans were accurate. During the lumbar spine scan, participants were placed in the supine position and a foam block was placed underneath their knees/calf to ensure their spine was flat on the scanner bed [51]. During the femoral neck scan, a triangular block was placed between the participants legs to achieve an internal rotation preventing the lesser trochanter from influencing the BMD [51]. All scans were conducted by the same Queensland Radiation Health licenced researcher (C.L.) using the predetermined mode by the auto scan feature in the software. Coefficients of variants (CV) for the lab have been reported elsewhere [52] reporting, a CV for BMD of 0.5–1.5%. Participants were grouped based on their BMD results according to the World Health Organisation into the following groups: normal BMD (T-score of > − 1.5), osteopenia (T-score between − 1.5 and − 2.5), and osteoporotic (T-score < − 2.5) [52].

### Socio-demographic and physical activity

Participants completed an online survey gathering socio-demographic information including age, gender, marital status, and income, administered via Qualtrics^®^. Leisure time physical activity frequency was assessed with the Godin Leisure-Time questionnaire [53]. The Activities-Specific Balance Confidence (ABC) scale was completed to assess falls confidence [54].

### Statistical analysis

Normality of data distribution was assessed via Shapiro–Wilk and measures of skewness and kurtosis. Data that were not normally distributed were transformed using the natural Log or Log base 10 and normality was tested again. All continuous variables are expressed as means ± standard deviation and categorical data are expressed as frequencies and percentages. Participants were grouped based on DII score into pro- or anti-inflammatory dietary groups and BMD T-score indicative of osteoporosis classification. A one-way ANOVA was used to compare participant characteristic data and BMD between groups. Chi-square tests examined between group difference for categorical data and the Fishers exact value was reported in sample sizes less than n < 5. Non-parametric tests were conducted for variables that were not normally distributed post transformation. Multiple linear regression analyses were used to explore associations between DII, lumbar spine BMD and wards triangle BMD controlling for the following covariates: age, gender, comorbidities, waist circumference, physical activity levels, calcification, medication, supplements, and energy intake (kJ). Dietary data were further explored by investigating associations between key nutrients and food groups (energy, protein, polyunsaturated fat, calcium, potassium, vegetables and legumes/beans, fruit, dairy products and lean meats and alternatives) known to influence bone density and BMD accounting for the prementioned covariates [21, 22, 55]. A calcification score accounts for developed mineralisation in paravertebral structures and was applied following the Orwoll et al. method [56]. All data were analysed using Microsoft Excel 2019 and SPSS (version 22.0, SPSS, Inc., Chicago, IL, USA) with significance set at p < 0.05.

## Results

A total of 135 participants were recruited, however, after excluding under- (n = 21) and over-reporters (n = 1) and those completing only one 24 h recall (n = 19) the final analysis included n = 94 community-dwelling older adults. Most participants had a healthy body weight, were sufficiently active, were married or living with a partner, completed tertiary education, and there were no participants who smoked cigarettes (Table 1). A total of six participants reported being diagnosed with osteoporosis by a medical professional and most participants (61.7%) reported following an anti-inflammatory diet.

**Table 1:** Participant characteristics of total group and grouped according to dietary inflammatory index score, n = 94

	Total	Dietary inflammatory index group		p-value^b^
		Anti-inflammatory diet (n = 58)	Pro-inflammatory diet (n = 36)	
Participant characteristics				
Age (years)	72.88 ± 4.9	72.55 ± 4.7	73.41 ± 5.3	0.414
Female n (%)	72 (76.6)	42 (44.68)	30 (31.91)	0.317
Weight (kg)	70.34 ± 11.8	72.30 ± 11.9	67.18 ± 11.1	0.041
Height (m)	1.66 ± 0.1	1.67 ± 0.1	1.63 ± 0.1	0.005
BMI (kg/m^2^)	25.55 ± 3.7	25.76 ± 3.9	25.21 ± 3.5	0.491
Waist circumference (cm)	86.33 ± 11.7	87.18 ± 11.6	84.95 ± 11.8	0.370
2 or more co-morbidities n (%)	12 (12.8)	9 (9.6)	3 (3.2)	0.363
Leisure time exercise n (%)				0.522
Insufficiently/moderately active	11 (11.7)	8 (8.5)	3 (3.2)	
Active	83 (88.3)	50 (53.2)	33 (35.1)	
Marital status n (%)				0.027
Married or partnered	63 (97.0)	39 (41.5)	20 (21.3)	
Single/widowed	14 (14.9)	4 (4.3)	10 (10.6)	
Separated or divorced	15 (16.0)	10 (10.6)	5 (5.3)	
Highest level of education n (%)				0.458
Primary/secondary education	22 (23.4)	15 (16.0)	7 (7.4)	
Vocational education	8 (8.5)	6 (6.4)	2 (2.1)	
Tertiary education	61 (64.9)	36 (38.3)	25 (26.6)	
Household income n (%) [57]^a^				0.387
Lowest income	35 (37.2)	18 (20.2)	17 (18.1)	
Low income	25 (26.6)	16 (17.0)	9 (9.6)	
Middle/high income	19 (20.7)	13 (13.8)	6 (6.4)	
Undisclosed	13 (14.1)	10 (10.6)	3 (3.2)	
BMD and falls confidence				
Lumbar spine L1–L4 (g/cm^2^)	1.15 ± 0.2	1.13 ± 0.2	1.19 ± 0.2	0.163
Wards triangle (g/cm^2^)^c^	0.65 ± 0.1	0.65 ± 0.1	0.64 ± 0.1	0.680
Falls confidence (%)	91.32 ± 8.0	91.98 ± 7.8	90.24 ± 8.3	0.309

^a^Australian Dollars

^b^T-test analysis compared DII groups for quantitative variables and Chi-square tests for qualitative variables, Fishers exact has been used for variables where n < 5

^c^Missing data n = 92

Multiple linear regression analysis findings are depicted in Table 2 illustrating no association between DII, lumbar spine, or femoral neck (wards triangle) BMD.

**Table 2:** Multiple linear regression coefficients expressing associations between bone mineral density and dietary inflammatory index

Unadjusted model		Model 1^a^		Model 2^b^		Model 3^c^	
Β (95%CI)	*p* Value	Β (95%CI)	*p* Value	Β (95%CI)	*p* Value	Β (95%CI)	*p* Value
Lumbar spine L1–L4 (g/cm^2^)							
0.020 (− 0.008, 0.047)	0.155	0.022 (− 0.005, 0.050)	0.104	0.022 (− 0.007, 0.051)	0.128	0.021 (− 0.008, 0.051)	0.155
Wards triangle (g/cm^2^)							
− 0.001 (− 0.019, 0.016)	0.866	0.005 (− 0.011, 0.021)	0.560	0.005 (− 0.012, 0.022)	0.564	0.005 (− 0.012, 0.022)	0.555

*DII* dietary inflammatory index

^a^Adjusted for calcification, supplements, age and gender

^b^Adjusted for calcification, energy intake, supplements, age, gender, waist circumference

^c^Adjusted for calcification, energy intake, medication, supplements, age, gender, waist circumference and physical activity level

Table 3 presents the results for DII score, energy, nutrient, and food group intake of the total group and stratified according to BMD T-score. A total of 75.6% (n = 81) of participants were identified with osteopenia or osteoporosis. There were no differences in energy, nutrient, or food group intake between groups with the exception of alcoholic beverages (p = 0.014) and a higher intake of lean meats in the osteoporosis sub-group (p = 0.027).

**Table 3:** Nutrient and food group intake of total group and stratified by bone mineral density T-Score

	Total group (n = 94)	Normal BMD^a^ (n = 23)	Osteopenia (n = 45)	Osteoporosis (n = 26)	p-Value^b^
DII	− 0.50 ± 1.6	− 0.75 ± 1.6	− 0.28 ± 1.6	− 0.68 ± 1.6	0.429
Energy (kJ)	7794.56 ± 1595.4	7927.92 ± 1452.6	7926.63 ± 1702.3	7967.41 ± 1553.4	0.622
Macro-nutrients					
Protein (g)	80.77 ± 19.5	79.22 ± 14.0	78.99 ± 21.7	85.22 ± 19.7	0.487
Protein (g/kg body weight)	1.17 ± 0.3	1.07 ± 0.2	1.17 ± 0.3	1.24 ± 0.3	0.105
Carbohydrate (g)	177.44 ± 53.0	181.02 ± 41.4	172.51 ± 53.8	182.81 ± 61.3	0.688
Fibre (g)	25.91 ± 8.0	25.65 ± 6.4	26.48 ± 8.4	25.17 ± 8.8	0.624
Total fat (g)	76.51 ± 21.5	73.53 ± 17.4	77.81 ± 23.7	76.90 ± 21.1	0.739
Saturated fat (g)	29.87 ± 10.0	29.47 ± 7.8	30.46 ± 11.1	29.20 ± 10.2	0.984
Monounsaturated fat (g)	28.63 ± 8.7	26.83 ± 8.4	29.04 ± 8.7	29.51 ± 9.0	0.512
Polyunsaturated fat (g)	11.34 ± 5.0	10.47 ± 3.0	11.49 ± 6.1	11.86 ± 4.3	0.523
Micro-nutrients					
Omega-3 (g)	2.26 ± 1.1	2.43 ± 1.0	2.25 ± 1.3	2.13 ± 0.8	0.565
Calcium (mg)	847.31 ± 257.0	863.94 ± 268.7	847.63 ± 249.1	932.04 ± 269.2	0.771
Potassium (mg)	3242.49 ± 845.9	3308.58 ± 766.9	3183.02 ± 940.2	3290.59 ± 758.9	0.595
Magnesium (mg)	429.43 ± 768.9	338.03 ± 56.3	346.48 ± 107.3	366.12 ± 86.4	0.305
Vitamin C (mg)	115.91 ± 74.5	142.92 ± 104.9	102.09 ± 60.4	115.94 ± 59.3	0.290
Vitamin B12 (µg)	4.16 ± 1.9	4.23 ± 1.3	3.98 ± `1.6	4.42 ± 2.7	0.542
Daily average food group and food intake^c^					
Grain (cereal) foods, mostly wholegrain and/or high cereal fibre varieties (serves)	4.84 ± 2.1	4.31 ± 1.7	5.00 ± 2.2	5.02 ± 2.3	0.464
Refined grain foods (serves)	3.24 ± 2.1	3.11 ± 1.8	3.19 ± 2.2	3.44 ± 2.1	0.791
Wholegrain foods (serves)	1.60 ± 1.3	1.20 ± 0.9	1.81 ± 1.4	1.57 ± 1.6	0.162
Vegetables and legumes/beans (serves)	5.08 ± 2.8	5.17 ± 2.9	5.26 ± 2.8	4.69 ± 2.8	0.596
Fruit (serves)	1.36 ± 0.8	1.34 ± 0.9	1.29 ± 0.8	1.49 ± 0.8	0.570
Milk, yoghurt, cheese and/or other alternatives, mostly reduced fat (serves)	1.73 ± 0.8	1.78 ± 0.8	1.75 ± 0.8	1.64 ± 0.8	0.783
Lean meats and poultry, fish, eggs, tofu, nuts, and seeds. (serves)	1.97 ± 0.9	1.70 ± 0.7	1.90 ± 1.1	2.32 ± 0.8	0.027
Processed meats (serves)	0.22 ± 0.4	0.29 ± 0.3	0.18 ± 0.2	0.24 ± 0.5	0.444
Alcoholic drinks (standard drink^d^)	1.34 ± 1.6	2.11 ± 1.8	0.95 ± 1.1	1.34 ± 1.8	0.014
Solid fat equivalents (tsp)	9.16 ± 3.7	9.39 ± 3.0	9.27 ± 3.8	8.77 ± 4.1	0.819
Added sugar (tsp)	25.06 ± 17.4	26.35 ± 13.1	22.08 ± 18.0	29.07 ± 19.0	0.244

^a^Cut-points determined by the World Health Organisation (normal BMD T score: > − 1.0, osteopenia T score: − 1.0 to − 2.5, osteoporosis T score: <−2.5) [58]

^b^One−way ANOVA compared E-DII groups for normally distributed quantitative variables and Kruskal–Wallis tests for data that was non normally distributed

^c^Food serves determined by the Australian Guide to Healthy Eating five core food groups [59]

^d^One standard drink = 10 g of alcohol

Multiple linear regression statistics explored the associations between BMD, nutrient, and food group intakes in Table 4. There was a significant negative association between the *lean meat and alternative* food group and femoral neck BMD (g/cm^2^) in adjusted models.

**Table 4:** Multiple linear regression coefficients expressing associations between nutrients, food groups and bone mineral density

	Unadjusted model		Model 1^a^		Model 2^b^		Model 3^c^	
	Β (95%CI)	*p* Value	Β (95%CI)	*p* Value	Β (95%CI)	*p* Value	Β (95%CI)	*p* Value
Lumbar spine L1–L4 (g/cm^2^)								
Energy	0.000 (0.000, 0.000)	0.816	− 0.000 (0.000, 0.000)	0.415	− 0.000 (0.000, 0.000)	0.305	− 0.000 (0.000, 0.000)	0.364
Macro-nutrients								
Protein	− 0.001 (− 0.003, 0.002)	0.619	− 0.002 (− 0.004, 0.001)	0.155	− 0.002 (− 0.004, 0.001)	0.168	− 0.002 (− 0.004, 0.001)	0.170
Polyunsaturated fat	0.000 (− 0.009, 0.008)	0.917	− 0.001 (− 0.009, 0.008)	0.898	− 0.001 (− 0.010, 0.008)	0.857	− 0.001 (− 0.010, 0.008)	0.853
Micro-nutrients								
Calcium	0.000 (0.000, 0.000)	0.237	0.000 (0.000, 0.000)	0.419	0.000 (0.000, 0.000)	0.451	0.000 (0.000, 0.000)	0.305
Potassium	− 0.000 (0.000, 0.000)	0.958	− 0.000 (0.000, 0.000)	0.315	− 0.000 (0.000, 0.000)	0.271	0.000 (0.000, 0.000)	0.341
Food groups								
Vegetables and legumes/beans	− 0.007 (− 0.023, 0.008)	0.349	− 0.007 (− 0.023, 0.009)	0.373	− 0.008 (− 0.023, 0.008)	0.328	− 0.007 (− 0.023, 0.008)	0.355
Fruit	− 0.018 (− 0.073, 0.038)	0.522	− 0.030 (− 0.087, 0.027)	0.300	− 0.027 (− 0.086, 0.031)	0.352	− 0.024 (− 0.082, 0.035)	0.428
Dairy products	0.047 (− 0.009, 0.103)	0.099	0.039 (− 0.018, 0.096)	0.181	0.037 (− 0.021, 0.095)	0.208	0.043 (− 0.015, 0.102)	0.144
Lean meat and alternatives	− 0.163 (− 0.084, 0.010)	0.117	− 0.205 (− 0.094, 0.000)	0.051	− 0.199 (− 0.093, 0.002)	0.060	− 0.196 (− 0.094, 0.003)	0.068
Femoral neck (g/cm^2^)								
Energy	0.000 (0.000, 0.000)	0.138	− 0.000 (0.000, 0.000)	0.911	− 0.000 (0.000, 0.000)	0.461	− 0.000 (0.000, 0.000)	0.380
Macro-nutrients								
Protein	0.000 (− 0.001, 0.002)	0.748	− 0.001 (− 0.002, 0.001)	0.201	− 0.001 (− 0.003, 0.000)	0.135	− 0.001 (− 0.003, 0.000)	0.129
Polyunsaturated fat	− 0.002 (− 0.008, 0.003)	0.440	− 0.003 (− 0.008, 0.002)	0.265	− 0.003 (− 0.008, 0.002)	0.206	− 0.003 (− 0.008, 0.002)	0.210
Micro-nutrients								
Calcium	0.000 (0.000, 0.000)	0.368	0.000 (0.000, 0.000)	0.816	0.000 (0.000, 0.000)	0.870	− 0.000 (0.000, 0.000)	0.976
Potassium	0.000 (0.000, 0.000)	0.202	0.000 (0.000, 0.000)	0.970	0.000 (0.000, 0.000)	0.781	0.000 (0.000, 0.000)	0.642
Food groups								
Vegetables and legumes/beans	0.005 (− 0.004, 0.015)	0.274	0.004 (− 0.005, 0.014)	0.341	0.003 (− 0.006, 0.013)	0.456	0.003 (− 0.006, 0.012)	0.489
Fruit	− 0.017 (− 0.051, 0.018)	0.333	− 0.013 (− 0.047, 0.020)	0.435	− 0.008 (− 0.042, 0.025)	0.616	− 0.012 (− 0.045, 0.022)	0.496
Dairy products	0.020 (− 0.015, 0.055)	0.250	0.013 (− 0.020, 0.046)	0.441	0.011 (− 0.022, 0.044)	0.507	0.009 (− 0.025, 0.042)	0.608
Lean meat and alternatives	− 0.168 (− 0.054, 0.006)	0.110	− 0.251 (− 0.064, − 0.008)	0.011*	− 0.233 (− 0.062, − 0.006)	0.014	− 0.229 (− 0.061, − 0.005)	0.023*

* indicates *p* < 0.05

^a^Adjusted for calcification, age and gender

^b^Adjusted for calcification, age, gender, waist circumference

^c^Adjusted for calcification, medication, supplements, age, gender, waist circumference and physical activity level

## Discussion

This study investigated the association between dietary inflammation, dietary intake, and BMD in community-dwelling older adults. Key findings showed the majority of community-dwelling older adults followed an anti-inflammatory diet, almost half presented with osteopenia, and more than a quarter with osteoporosis. No association was found between DII, spine or femoral neck BMD but the *lean meat and alternative* food group was negatively associated with femoral neck BMD in adjusted models.

Cross-sectional studies report inconsistent findings on the associations between dietary inflammation and BMD [32–34, 36–38]. Song et al. [37] reported no significant difference in BMD at all sites among post-menopausal women following an anti-inflammatory diet compared to those following a pro-inflammatory diet, which is consistent with present findings. In addition, Jackson et al. [32] found no significant associations between E-DII and spine or total hip BMD in post-menopausal women. In contrast, Shivappa et al. [36] found a beneficial association between an anti-inflammatory diet and spine BMD, but not femoral neck BMD, in a cohort of post-menopausal women. Whilst Li et al. [33] found significant associations between spine and total hip BMD with E-DII in post-menopausal women, as well as greater total leg BMD in those that followed an anti-inflammatory diet compared to those who followed a pro-inflammatory diet (p < 0.05) [33]. Middle-aged adults that followed an anti-inflammatory diet had significantly higher femoral neck and spine BMD compared to those following a pro-inflammatory diet [34]. Zhao et al. [38] found a positive association between DII and BMD, however BMD sites differed between men and women. Femoral neck and total hip BMD was associated with DII in older women while spine BMD was associated with DII in older men but not in women [38]. These findings contrast with the current study where no association was found between DII and BMD at spine and femoral neck sites. Sub-group analysis based on sex was not possible due to small sample size in the current study. A relationship between DII and BMD could be likely given that chronic low-grade inflammation is known to negatively impact BMD later in life [11]. Bone turnover markers, that may be predictive of osteoporosis, are more sensitive to change with heightened inflammation [60]. Inflammation affects specific bone turnover markers such as C-terminal telopeptide (CTX), a marker of bone resorption, and procollagen type I N-terminal propeptide (P1NP), which indicates bone formation [14, 60]. Increased levels of inflammatory cytokines can alter the dynamics of these markers, contributing to accelerated bone turnover and a loss in BMD [14]. It is also known that increases in pro-inflammatory cytokines are associated with ageing, and augment myelopoiesis and osteoclastogenesis, which may contribute to the decline in BMD in later years [11]. The current study did not include measurement of inflammatory cytokines, limiting the ability to draw definitive conclusions about whether changes in BMD are influenced by inflammatory cytokine concentrations. This is a notable gap in the literature, and future research should measure inflammatory cytokines to better understand the mechanisms that may connect DII with BMD.

Longitudinal studies report mixed results on the beneficial relationship between following an anti-inflammatory diet and greater BMD. Over six years, BMD loss was significantly reduced in adults that followed an anti-inflammatory diet compared to those that followed a pro-inflammatory diet [35]. Similarly, in a 10-year longitudinal study, an independent beneficial association between E-DII and total hip and lumbar spine BMD in men but not in women in fully adjusted models was reported [31]. The authors concluded other risk factors such as hormonal changes in women may partly explain the lack of association between dietary inflammation and BMD. Current evidence supports the conclusion that BMD loss is influenced by the reduction of estrogen in postmenopausal women [61]. The influence of hormonal changes on BMD over time which may diminish the effects of following an anti-inflammatory diet post menopause [61]. Therefore, promoting an anti-inflammatory diet earlier in life, especially among women, could potentially mitigate bone loss and preserve BMD before menopause onset and the associated change in BMD due to hormonal status however, further research is warranted in this area.

Several factors may contribute to the inconsistency of findings on the relationship between dietary inflammation and BMD. Non-modifiable factors such as age and gender play a significant role in bone health. Trabecular bone loss begins at the age of 50 years where a linear decline in BMD is common for both men and women however, the annual decline in women (average rate ~ 28%) is greater than that of men (average rate ~ 18%) [6]. During menopausal onset there is an increase rate in bone turnover with bone resorption increasing by ~ 90% and bone formation ~ 45% resulting in a net loss of BMD. This change in bone turnover is contributed to the hormonal changes associated with the menopausal transition period [62]. These hormonal changes seen in post-menopausal women may partly contribute to mixed findings in the literature on the association between dietary inflammation and bone health [32, 33, 35–37]. Moreover, it is a likely reason for the lack of an association between DII an BMD in the current study since most participants were post-menopausal women [31, 32, 37].

Of interest was a significant difference in marital status between those following an anti-inflammatory diet compared to a pro-inflammatory diet in that more people who were single or widowed followed a pro-inflammatory diet. The regular consumption of perishables such as fresh fruits and vegetables have been reported to be lower in single-person households whilst more takeaway meals are consumed compared to households with two or more individuals [63, 64]. Thereby consuming less foods with anti-inflammatory properties and more food with pro-inflammatory ingredients. These findings highlight a unique opportunity to improve dietary choices of older adults living alone to mitigate the potential effect of poor food choice, providing a novel area for future research.

Despite the known relationship between key nutrients and food groups (energy, protein, polyunsaturated fat, calcium, potassium, vegetables and legumes/beans, fruit, dairy products and lean meats and alternatives) [21, 22, 55] the current study found no relationship between key nutrients and food groups with BMD in unadjusted models. Furthermore, no differences in nutrient intake or food groups were found when participants were stratified according to BMD T-scores. There was a trend in higher intake of *lean meat and alternatives* food groups in those identified with osteoporosis. A negative association was found between femoral neck BMD and the *lean meat and alternatives* food group in adjusted models. Several studies have explored the relationship between protein intake and BMD suggesting a positive association between a higher protein intake and bone health in older adults, however the certainty in the evidence is low [65–67]. Moreover, there is debate in the literature on the effect of plant versus animal protein foods on BMD. High quality research studies are needed to further the understanding of the complex interplay between the source of dietary protein and bone health in older adults.

The current study included a relatively healthy sample of older adults compared to most older Australian adults with the majority being sufficiently active, within a healthy body range, and only a few suffering from comorbidities [68, 69]. It is worth noting a high number of participants were identified with either osteopenia or osteoporosis, which may suggest a sample with compromised bone health. However, this proportion with low BMD is not too dissimilar to that of the Australian national average, where approximately 66% of adults are reported to have osteopenia or osteoporosis [70]. This notable prevalence of participants exhibiting low BMD may also suggest an elevated susceptibility to fractures; however, it is pertinent to highlight findings from a subset of this study which explored the associations between the DII and measures of sarcopenia symptomology [57]. Within this subset, it was observed that the strength and mobility levels of this cohort were notably high [57]. Despite approximately two-thirds of individuals of the current study exhibiting low bone density, their fracture risk is likely low due to functional ability and muscle strength, as evidenced in prior research [57].

### Strengths and limitations

The inclusion of 31 out of 45 potential dietary parameters in the calculation of the DII score may be viewed as a limitation of the present study. However, Hébert et al. [71] reported the exclusion of similar dietary parameters in calculating the DII score does not reduce the validity of the calculation. Similar to others [34, 36, 38, 48], the exclusion of garlic, ginger, saffron, turmeric, pepper, thyme/oregano, and rosemary were due to quantification difficulty, while vitamin D, flavan-3-ol, flavones, flavanols, flavanones, anthocyanidins and isoflavones were not calculated due to food analysis software package constraints. Moreover, we recognise limitations inherent in the DII calculation itself such as not accounting for individual supplement use or data skewing through the consumption of nutrient/energy dense foods [72]. While these limitations are inherent to the tool itself, it is crucial to acknowledge them as constraints in this present study. Using the multiple pass method to collect dietary data on habitual dietary intake is a strength of this study [41]. Additionally, the inclusion of three 24-h dietary recalls compared to one timepoint improves the validity of the data as a representation of habitual dietary intake at the time of the study, further bolstering the reliability of the findings. There are strengths and limitations with all dietary recall methods in particularly limitations associated with retrospective methods such as 24-h dietary recalls, however, it is recommended to complete a minimum of three 24-h recalls following the multiple pass method to estimate people’s usual energy intake because of the substantial intraindividual, day-to-day variation that occurs in food intake [73]. Whilst excluding those deemed to be under-reporting following the Goldberg method is seen as a strength of the present study, it is acknowledged that estimations using the Mifflin-St Jeor formula, as well as with any other formulas, may not provide an accurate representation of an individual’s basal metabolic rate [45]. Given the small sample size of the present study, caution is advised when interpreting the results as a type II error may occur with small sample sizes. Consequently, significant differences between groups may not have been detected due to low power. Finally, it is important to note that the cross-sectional study design doesn't allow for conclusions regarding the causality between dietary inflammation and BMD.

## Conclusion

In this cohort of apparently healthy and sufficiently active older adults, dietary inflammation was not associated with BMD at the spine and femoral neck despite a large proportion identified with low BMD. It is acknowledged that other factors might override the effect of dietary inflammation on BMD in healthy, active older adults. The findings underscore the complex synergistic effect of modifiable and non-modifiable factors such as diet, exercise, age, and gender have on BMD in physically active community-dwelling older adults. Longitudinal studies examining the predictive value of following an anti-inflammatory diet on BMD over the course of adulthood is needed to advance the understanding of dietary inflammation and BMD in older adults.

## Data Availability

Data supporting reported results are available upon reasonable request and in accordance with the ethical principles.

## References

[1] Kanis J (2007) Assessment of osteoporosis at the primary health-care level. In: Group WHOS (ed)

[2] Martin A, Sornay-Rendu E, Chandler J, Duboeuf F, Girman C, Delmas P (2002) The impact of osteoporosis on quality-of-life: the OFELY cohort. Bone 31(1):32–36 12110409 10.1016/s8756-3282(02)00787-1

[3] Kemmak AR, Rezapour A, Jahangiri R, Nikjoo S, Farabi H, Soleimanpour S (2020) Economic burden of osteoporosis in the world: a systematic review. Med J Islam Repub Iran 34:154 33437750 10.34171/mjiri.34.154 PMC7787041

[4] Gullberg B, Johnell O, Kanis J (1997) World-wide projections for hip fracture. Osteoporos Int 7:407–413 9425497 10.1007/pl00004148

[5] Manolagas SC (2000) Birth and death of bone cells: basic regulatory mechanisms and implications for the pathogenesis and treatment of osteoporosis. Endocr Rev 21(2):115–137 10782361 10.1210/edrv.21.2.0395

[6] Drake MT, Clarke BL, Lewiecki EM (2015) The pathophysiology and treatment of osteoporosis. Clin Ther 37(8):1837–1850 26163201 10.1016/j.clinthera.2015.06.006

[7] Hendrickx G, Boudin E, Van Hul W (2015) A look behind the scenes: the risk and pathogenesis of primary osteoporosis. Nat Rev Rheumatol 11(8):462–474 25900210 10.1038/nrrheum.2015.48

[8] Jilka RL, O’Brien CA, Roberson PK, Bonewald LF, Weinstein RS, Manolagas SC (2014) Dysapoptosis of osteoblasts and osteocytes increases cancellous bone formation but exaggerates cortical porosity with age. J Bone Miner Res 29(1):103–117 23761243 10.1002/jbmr.2007 PMC3823639

[9] Zhang Q, Cai W, Wang G, Shen X (2020) Prevalence and contributing factors of osteoporosis in the elderly over 70 years old: an epidemiological study of several community health centers in Shanghai. Ann Palliative Medicine 9(2):23138–23238 10.21037/apm.2020.02.09 32156135

[10] Lacativa PGS, Farias MLFD (2010) Osteoporosis and inflammation. Arquivos Brasileiros de Endocrinologia Metabologia 54:123–132 20485900 10.1590/s0004-27302010000200007

[11] Abdelmagid SM, Barbe MF, Safadi FF (2015) Role of inflammation in the aging bones. Life Sci 123:25–34 25510309 10.1016/j.lfs.2014.11.011

[12] Moshage H (1997) Cytokines and the hepatic acute phase response. J Pathol A J Pathol Soc G B Irel 181(3):257–266 10.1002/(SICI)1096-9896(199703)181:3<257::AID-PATH756>3.0.CO;2-U 9155709

[13] Ding C, Parameswaran V, Udayan R, Burgess J, Jones G (2008) Circulating levels of inflammatory markers predict change in bone mineral density and resorption in older adults: a longitudinal study. J Clin Endocrinol Metab 93(5):1952–1958 18285417 10.1210/jc.2007-2325

[14] Epsley S, Tadros S, Farid A, Kargilis D, Mehta S, Rajapakse CS (2021) The effect of inflammation on bone. Front Physiol 11:511799 33584321 10.3389/fphys.2020.511799 PMC7874051

[15] Benedetti MG, Furlini G, Zati A, Letizia MG (2018) The effectiveness of physical exercise on bone density in osteoporotic patients. Biomed Res Int 2018(1):4840531 30671455 10.1155/2018/4840531 PMC6323511

[16] Leeuwenburgh C, Heinecke J (2001) Oxidative stress and antioxidants in exercise. Curr Med Chem 8(7):829–838 11375753 10.2174/0929867013372896

[17] Ozcivici E, Luu YK, Adler B, Qin Y-X, Rubin J, Judex S et al (2010) Mechanical signals as anabolic agents in bone. Nat Rev Rheumatol 6(1):50–59 20046206 10.1038/nrrheum.2009.239 PMC3743048

[18] Prentice A (2004) Diet, nutrition and the prevention of osteoporosis. Public Health Nutr 7(1a):227–243 14972062 10.1079/phn2003590

[19] Sözen T, Özışık L, Başaran NÇ (2017) An overview and management of osteoporosis. Eur J Rheumatol 4(1):46 28293453 10.5152/eurjrheum.2016.048 PMC5335887

[20] Martiniakova M, Babikova M, Mondockova V, Blahova J, Kovacova V, Omelka R (2022) The role of macronutrients, micronutrients and flavonoid polyphenols in the prevention and treatment of osteoporosis. J Nutr 14(3):523 10.3390/nu14030523 PMC8839902 35276879

[21] Sahni S, Mangano KM, McLean RR, Hannan MT, Kiel DP (2015) Dietary approaches for bone health: lessons from the Framingham Osteoporosis Study. Curr Osteoporos Rep 13:245–255 26045228 10.1007/s11914-015-0272-1 PMC4928581

[22] Uusi-Rasi K, Kärkkäinen MU, Lamberg-Allardt CJ (2013) Calcium intake in health maintenance–a systematic review. Food Nutr Res 57(1):21082 10.3402/fnr.v57i0.21082 PMC3657072 23687486

[23] Muñoz-Garach A, García-Fontana B, Muñoz-Torres M (2020) Nutrients and dietary patterns related to osteoporosis. J Nutr 12(7):1986 10.3390/nu12071986 PMC7400143 32635394

[24] Aiello A, Accardi G, Candore G, Carruba G, Davinelli S, Passarino G et al (2016) Nutrigerontology: a key for achieving successful ageing and longevity. Springer, Berlin, pp 1–5 10.1186/s12979-016-0071-2 PMC4875663 27213002

[25] Tapsell LC, Neale EP, Satija A, Hu FB (2016) Foods, nutrients, and dietary patterns: interconnections and implications for dietary guidelines. Adv Nutr 7(3):445–454 27184272 10.3945/an.115.011718 PMC4863273

[26] Hu FB (2002) Dietary pattern analysis: a new direction in nutritional epidemiology. Curr Opin Lipidol 13(1):3–9 11790957 10.1097/00041433-200202000-00002

[27] Zhong GC, Wang K, Peng Y, Shivappa N, Hebert JR, Wu YQL et al (2020) Dietary inflammatory index and incidence of and death from primary liver cancer: a prospective study of 103,902 American adults. Int J Cancer 147(4):1050–1058 32142166 10.1002/ijc.32954

[28] Anderson AL, Harris TB, Tylavsky FA, Perry SE, Houston DK, Lee JS et al (2012) Dietary patterns, insulin sensitivity and inflammation in older adults. Eur J Clin Nutr 66(1):18–24 21915138 10.1038/ejcn.2011.162 PMC3251708

[29] Cavicchia PP, Steck SE, Hurley TG, Hussey JR, Ma Y, Ockene IS et al (2009) A new dietary inflammatory index predicts interval changes in serum high-sensitivity C-reactive protein. J Nutr 139(12):2365–2372 19864399 10.3945/jn.109.114025 PMC2777480

[30] Esmaillzadeh A, Kimiagar M, Mehrabi Y, Azadbakht L, Hu FB, Willett WC (2007) Dietary patterns and markers of systemic inflammation among Iranian women. J Nutr 137(4):992–998 17374666 10.1093/jn/137.4.992

[31] Cervo MM, Shivappa N, Hebert JR, Oddy WH, Winzenberg T, Balogun S et al (2020) Longitudinal associations between dietary inflammatory index and musculoskeletal health in community-dwelling older adults. Clin Nutr 39(2):516–523 30852031 10.1016/j.clnu.2019.02.031

[32] Jackson MK, Bilek LD, Waltman NL, Ma J, Hébert JR, Price S et al (2023) Dietary inflammatory potential and bone outcomes in midwestern post-menopausal women. J Nutr 15(19):4277 10.3390/nu15194277 PMC10574295 37836561

[33] Li R, Zhan W, Huang X, Wang J, Lv S, Liang L et al (2022) Associations between dietary inflammatory index (DII) and bone health among postmenopausal women in the United States. Int J Gynecol Obstet 158(3):663–670 10.1002/ijgo.14024 34767250

[34] Mazidi M, Shivappa N, Wirth M, Hebert J, Vatanparast H, Kengne A (2017) The association between dietary inflammatory properties and bone mineral density and risk of fracture in US adults. Eur J Clin Nutr 71(11):1273–1277 29019343 10.1038/ejcn.2017.133

[35] Orchard T, Yildiz V, Steck SE, Hébert JR, Ma Y, Cauley JA et al (2017) Dietary inflammatory index, bone mineral density, and risk of fracture in postmenopausal women: results from the women’s health initiative. JBMR 32(5):1136–1146 10.1002/jbmr.3070 PMC5413384 28019686

[36] Shivappa N, Hébert JR, Karamati M, Shariati-Bafghi S-E, Rashidkhani B (2016) Increased inflammatory potential of diet is associated with bone mineral density among postmenopausal women in Iran. Eur J Clin Nutr 55:561–568 10.1007/s00394-015-0875-4 25778389

[37] Song D, Kim J, Kang M, Park J, Lee H, Kim D-Y et al (2022) Association between the dietary inflammatory index and bone markers in postmenopausal women. PLoS ONE 17(3):e0265630 35298570 10.1371/journal.pone.0265630 PMC8929634

[38] Zhao S, Gao W, Li J, Sun M, Fang J, Tong L et al (2022) Dietary inflammatory index and osteoporosis: the National Health and Nutrition Examination Survey, 2017–2018. Endocrine 78(3):587–596 36044108 10.1007/s12020-022-03178-6

[39] Linton C, Wright HH, Wadsworth DP, Schaumberg MA (2022) Dietary inflammatory index and associations with sarcopenia symptomology in community-dwelling older adults. J Nutr 14(24):5319 10.3390/nu14245319 PMC9787040 36558478

[40] Norton K, Norton L (2011) Pre-exercise screening. Guide to the Australian adult pre-exercise screening system exercise and sports science Australia

[41] Moshfegh AJ, Rhodes DG, Baer DJ, Murayi T, Clemens JC, Rumpler WV et al (2008) The US Department of Agriculture automated multiple-pass method reduces bias in the collection of energy intakes. AJCN 88(2):324–332 10.1093/ajcn/88.2.324 18689367

[42] Zealand FSAaN (2024) AUSNUT 2011-13 food nutrient database: Food Standards Australia and New Zealand

[43] Black A (2000) The sensitivity and specificity of the Goldberg cut-off for EI: BMR for identifying diet reports of poor validity. Eur J Clin Nutr 54(5):395–404 10822286 10.1038/sj.ejcn.1600971

[44] Goldberg G, Black A, Jebb S, Cole T, Murgatroyd P, Coward W et al (1991) Critical evaluation of energy intake data using fundamental principles of energy physiology: 1. Derivation of cut-off limits to identify under-recording. Eur J Clin Nutr 45(12):569–581 1810719

[45] Mifflin MD, St Jeor ST, Hill LA, Scott BJ, Daugherty SA, Koh YO (1990) A new predictive equation for resting energy expenditure in healthy individuals. AJCN 51(2):241–247 10.1093/ajcn/51.2.241 2305711

[46] Shivappa N, Steck SE, Hurley TG, Hussey JR, Hébert JR (2014) Designing and developing a literature-derived, population-based dietary inflammatory index. Public Health Nutr 17(8):1689–1696 23941862 10.1017/S1368980013002115 PMC3925198

[47] Shivappa N, Steck SE, Hurley TG, Hussey JR, Ma Y, Ockene IS et al (2014) A population-based dietary inflammatory index predicts levels of C-reactive protein in the Seasonal Variation of Blood Cholesterol Study (SEASONS). Public Health Nutr 17(8):1825–1833 24107546 10.1017/S1368980013002565 PMC3983179

[48] Li S, Zeng M (2023) The association between dietary inflammation index and bone mineral density: results from the United States National Health and nutrition examination surveys. Ren Fail 45(1):2209200 37154137 10.1080/0886022X.2023.2209200 PMC10167883

[49] Winter JE, MacInnis RJ, Wattanapenpaiboon N, Nowson CA (2014) BMI and all-cause mortality in older adults: a meta-analysis. AJCN 99(4):875–890 10.3945/ajcn.113.068122 24452240

[50] Estrella-Castillo DF, Gómez-de-Regil L (2019) Comparison of body mass index range criteria and their association with cognition, functioning and depression: a cross-sectional study in Mexican older adults. BMC Geriatr 19:1–8 31795994 10.1186/s12877-019-1363-0 PMC6889317

[51] Nana A, Slater GJ, Hopkins WG, Burke LM (2012) Techniques for undertaking dual-energy X-ray absorptiometry whole-body scans to estimate body composition in tall and/or broad subjects. Int J Sport Nutr Exerc Metab 22(5):313–322 23011648 10.1123/ijsnem.22.5.313

[52] Nicholson VP, McKean MR, Slater GJ, Kerr A, Burkett BJ (2015) Low-load very high-repetition resistance training attenuates bone loss at the lumbar spine in active post-menopausal women. Calcif Tissue Int 96:490–499 25772806 10.1007/s00223-015-9976-6

[53] Godin G (2011) The Godin–Shephard leisure-time physical activity questionnaire. Health Fit J Can 4(1):18–22

[54] Powell LE, Myers AM (1995) The activities-specific balance confidence (ABC) scale. J Gerontol A Biol Sci Med Sci 50(1):M28–M34 10.1093/gerona/50a.1.m28 7814786

[55] Mithal A, Bonjour J-P, Boonen S, Burckhardt P, Degens H, El Hajj FG et al (2013) Impact of nutrition on muscle mass, strength, and performance in older adults. Osteoporos Int 24:1555–1566 23247327 10.1007/s00198-012-2236-y

[56] Orwoll ES, Oviatt SK, Mann T (1990) The impact of osteophytic and vascular calcifications on vertebral mineral density measurements in men. J Clin Endocrinol Metab 70(4):1202–1207 2318940 10.1210/jcem-70-4-1202

[57] Statistics Abo (2022) Household Income and Wealth, Australia. Australian Bureau of Statistics: Australian Government, Sydney

[58] Organisation WH (2003) Prevention and management of osteoporosis: WHO

[59] Goverment A. Eat for health—food groups

[60] Rosalki S (1998) Biochemical markers of bone turnover. Int J Clin Pract 52(4):255–256 9744151

[61] Sowers MR, Jannausch M, McConnell D, Little R, Greendale GA, Finkelstein JS et al (2006) Hormone predictors of bone mineral density changes during the menopausal transition. J Clin Endocrinol Metab 91(4):1261–1267 16403818 10.1210/jc.2005-1836

[62] Garnero P, Sornay-Rendu E, Chapuy MC, Delmas PD (1996) Increased bone turnover in late postmenopausal women is a major determinant of osteoporosis. JBMR 11(3):337–349 10.1002/jbmr.5650110307 8852944

[63] Moreau M, Plourde H, Hendrickson-Nelson M, Martin J (2015) Efficacy of nutrition education-based cooking workshops in community-dwelling adults aged 50 years and older. J Nutr Gerontol Geriatr 34(4):369–387 26571355 10.1080/21551197.2015.1084257

[64] Whitelock E, Ensaff H (2018) On your own: older adults’ food choice and dietary habits. J Nutr 10(4):413 10.3390/nu10040413 PMC5946198 29584644

[65] Bonjour J-P, Chevalley T, Amman P, Rizzoli R (2015) Protein intake and bone health. Springer, Berlin

[66] Sahni S, Broe KE, Tucker KL, McLean RR, Kiel DP, Cupples LA et al (2014) Association of total protein intake with bone mineral density and bone loss in men and women from the Framingham Offspring Study. Public Health Nutr 17(11):2570–2576 24168918 10.1017/S1368980013002875 PMC4103961

[67] Zittermann A, Schmidt A, Haardt J, Kalotai N, Lehmann A, Egert S et al (2023) Protein intake and bone health: an umbrella review of systematic reviews for the evidence-based guideline of the German Nutrition Society. Osteoporos Int 34(8):1335–1353 37126148 10.1007/s00198-023-06709-7 PMC10382330

[68] Goverment A (2018) Overweight and obesity. In: Welfare AIoHa (ed)

[69] Goverment A (2022) Physical Activity. Australian Bureau of Statistics, Sydney

[70] Watts J, Abimanyi-Ochom J, Sanders KM. Osteoporosis costing all Australian: a new burden of disease analysis-2012 to 2022. Deakin University; 2013.

[71] Hébert JR, Shivappa N, Wirth MD, Hussey JR, Hurley TG (2019) Perspective: the dietary inflammatory index (DII)—lessons learned, improvements made, and future directions. Adv Nutr 10(2):185–195 30615051 10.1093/advances/nmy071 PMC6416047

[72] Bahr LS, Franz K, Mähler A (2021) Assessing the (anti)-inflammatory potential of diets. Curr Opin Clin Nutr Metab Care 24(5):402–410 34155152 10.1097/MCO.0000000000000772

[73] Johnson RK (2002) Dietary intake—how do we measure what people are really eating? Obesity 10(s11):63S 10.1038/oby.2002.192 12446861

